# Newborn Parent Based Intervention to Increase Child Safety Seat Use

**DOI:** 10.3390/ijerph13080777

**Published:** 2016-08-02

**Authors:** Xiangxiang Liu, Jingzhen Yang, Fuyuan Cheng, Liping Li

**Affiliations:** 1 Injury Prevention Research Center, Medical College of Shantou University, 22 Xin Ling Road, Shantou 515041, China; xxliu2014@126.com (X.L.); cfyhcf@163.com (F.C.); 2 Center for Injury Research and Policy, The Research Institute at Nationwide Children’s Hospital, The Ohio State University, 700 Children’s Drive, Columbus, OH 43205, USA; Ginger.Yang@nationwidechildrens.org

**Keywords:** child passenger safety, CSS, newborn parents, intervention

## Abstract

This paper intends to assess the effect of a maternity department intervention on improvement of knowledge and use of child safety seats (CSS) among newborn parents. An intervention study included three groups (one education plus free CSS intervention group, one education only group, and one control group). The participants were parents of newborns in the maternity department of two hospitals. Both of the intervention groups received a folded pamphlet of child passenger safety, a height chart and standardized safety education during their hospital stay after giving birth. The education plus free CSS intervention group received an additional free CSS and professional installation training at hospital discharge. The control group received a pamphlet with educational information about nutrition and food safety. Three months after enrollment, a telephone follow-up was conducted among participants in the three groups. Data on child passenger safety knowledge, risky driving behaviors, and use of CSS were evaluated before and after the intervention. A total of 132 newborn parents were enrolled in the study; of those, 52 (39.4%) were assigned into the education plus free CSS intervention group, 44 (33.3%) were in the education intervention only group, and 36 (27.3%) were in the control group. No significant differences existed in demographics among the three groups. There was a significant difference in newborn parents’ child passenger safety knowledge and behaviors in the three groups before and after the intervention. In addition, the CSS use increased significantly in the education plus free CSS group after the intervention compared to parents in the education only or control groups. Education on safety, combined with a free CSS and professional installation training, were effective at increasing newborn parents’ knowledge and use of CSS. Future studies with larger sample sizes and longer follow-up are needed to determine a long-term effect of the intervention.

## 1. Introduction

Road traffic crashes are the leading cause of death and injury among children under 14 in China [1]. While child safety seats (CSS) have been used widely in western countries, the lack of national laws for CSS, coupled with poor parental knowledge about child passenger safety, result in a very low rate of CSS use in China [2]. Previous studies conducted in China showed that the rate of CSS use was about 5% in metropolitan areas [3,4], and less than 1% in a small city [2]. A more recent survey also showed that of 906 observed cars with infant passengers, only two (0.2%) infants were in a CSS [2]. The majority of newborns in China were held on laps by parents while traveling in a car to their homes following discharge from the hospital, putting newborns at potential risk [2].

Compulsory legislation can be effective to increase the use of CSS. Most high-income countries have a CSS law, while only a third of low- and middle-countries have CSS laws [5]. In the United States and other western countries, current guidelines recommend that all newborns discharged from hospitals be transported home in CSS, and that hospitals should have comprehensive policies and procedures in place for the discharge of newborns [6,7]. There is only one country in south-east Asia and four countries in the eastern Mediterranean that have CSS laws using the same criteria of western countries [5,8]. In 2000, Japan’s Road Traffic Law stipulating CSS use among children aged 0–5 years went into effect, while, in China, even today there is no national legislation [9]. To increase CSS use following hospital discharge and improve the knowledge of parents, many intervention programs have been implemented, including CSS education, CSS installation training, CSS discharge polices of newborns and CSS laws, all of which showed success in increasing CSS use [10,11,12,13,14,15,16,17,18,19]. A published review in 2001 showed that more than 10 programs included the strategies of providing free loaner CSS, giveaways or low-cost rentals to the parents of children, in addition to educational components such as instruction or written materials, professional installation training, or rehearsal of skills [20]. To date, only two intervention studies conducted in China have been published in the literature. One focused on education only to the birthing mothers in the maternity wards about the importance of CSS use [7]. Another used education along with free CSS to the parents of children ages three to eight [21]. The latter did not have a control group and only followed participants for six weeks. The results from both interventions showed that while parents increased knowledge on child passenger safety after intervention, CSS use was still very low. The results from these two studies call for more comprehensive intervention programs that combine education with free CSS and professional installation training in order to improve knowledge and behaviors of CSS use. We conducted a comprehensive intervention in maternity departments to parents of newborns. The purpose of this study was to determine the effect of the intervention on the improvement of knowledge of child passenger safety and use of CSS among parents with newborns. We hypothesized that parents assigned into the intervention group who received education and free CSS would have greater increases in child passenger safety knowledge and use CSS, as compared to parents in the education only or control groups.

## 2. Methods

### 2.1. Study Design and Participants 

An intervention study was conducted in two selected hospitals in Shantou, a coastal city located in China: (1) Shantou Women’s and Children’s Hospital, a general hospital equipped with 60 ward beds with about 300 infants delivered per month; and (2) Shantou University-affiliated Hospital, a general hospital equipped with 50 ward beds with 250 infants delivered per month. Both are non-profit hospitals. The parents of newborns from these two hospitals have similar demographic characteristics, such as language, income, educational background and occupation (*p* > 0.05). A convenience sample of parents of newborns was recruited to complete a questionnaire in person in the maternity wards after delivery. The inclusion criteria were: (1) the parent of a newborn; (2) both the parent and the newborn were healthy without any adverse postpartum complications; (3) owned a car; (4) had not bought a CSS; (5) lived in Shantou more than six months; (6) were able to read, write and speak Mandarin; and (7) agreed to participate through a signed consent document. The study protocol, along with the consent process, was approved by Medical Ethics Committees of Shantou University Medical College.

### 2.2. Ethical Statements

All subjects gave their informed consent for inclusion before they participated in the study. The study was conducted in accordance with the Declaration of Helsinki, and the protocol was approved by Medical Ethics Committees of Shantou University Medical College (Project identification code: SUMC-2015-39).

### 2.3. Intervention Type

Participating parents were systematically assigned into one of the three study conditions: 

Comparison group: Eligible participants who gave birth in Shantou University-affiliated Hospital were assigned to this group. Participating parents received a pamphlet that included standardized education about nutrition and food safety but not child passenger safety, and a height chart with no CSS information.

Education only intervention group: Eligible participants who gave birth in Shantou Women’s and Children’s Hospital in an odd month were assigned into this group. Participating parents received a folded pamphlet of child passenger safety, a height chart with knowledge, type, classification, and laws of CSS and standardized safety education during their hospital stay after giving birth.

Education plus free CSS intervention group: Eligible participants who gave birth in Shantou Women’s and Children’s Hospital in an even month were assigned into this group. Participating parents received a free convertible CSS suitable for a newborn, and professional installation training before they left the hospital, in addition to receiving the education materials that the education intervention only group received.

### 2.4. Intervention Data Collection and Survey Instruments

Two trained medicine graduates conducted the intervention. Before the intervention, the eligible participants were introduced to the research and were enrolled by the head nurse in the maternity department through signed informed consent. Following complete baseline assessment on child passenger safety knowledge, risky driving behaviors, and use of CSS, enrolled participants were then assigned into the three study conditions and received the respective intervention. Three months later, a phone interview was conducted among parents enrolled in the three groups. Data on child passenger safety knowledge, risky driving behaviors, and use of CSS were collected. Changes in knowledge and CSS use before and after the intervention were compared across three groups. The study was conducted from December 2014 to November 2015.

The survey instruments before and after the intervention were developed by the research team based on published work and the team’s previous work in this area. The questions asked included the following four parts: CSS use, child passenger safety knowledge, risky driving behavior, and socio-demographic characteristics. 

Use of CSS: The use of CSS was measured based on participating parents’ responses to the questions on whether or not they have used a CSS, the type of CSS, the installation position of CSS and intention of future use.

Child passenger safety knowledge: Seven items were included asking about parental knowledge about traffic safety, including seatbelts, airbags, holding a baby while in a moving car, feeding a baby in a moving car, use of CSS, the best suitable installation position, and CSS laws. 

Risky driving behaviors: Five survey items measured risky driving behaviors, including smoking while driving, drunk driving, using a mobile phone while driving, driver’s seatbelt use, and front passenger’s seatbelt use.

Demographic information collected included: gender, place of residence, degree of education of parents of newborn, profession of parents of newborns, and household per capita income.

### 2.5. Statistical Analysis

Survey data were entered into EpiData version 3.0 for Windows (EpiData Association, Odense, Denmark) and then analyzed using SPSS version 19.0 (SPSS Inc., Chicago, IL, USA). All the data was double-entered to ensure quality. The characteristics of participating parents were described using descriptive statistics. The differences in child passenger safety knowledge, risky driving behaviors, and CSS used were evaluated before and after the intervention using chi-square tests. Logistic regression was used to assess the effects of intervention on child passenger safety knowledge, risky driving behaviors, and CSS use. The significance level was set at ***α*** = 0.05.

## 3. Results

### 3.1. Characteristics of Participating Parents

**Figure 1:**
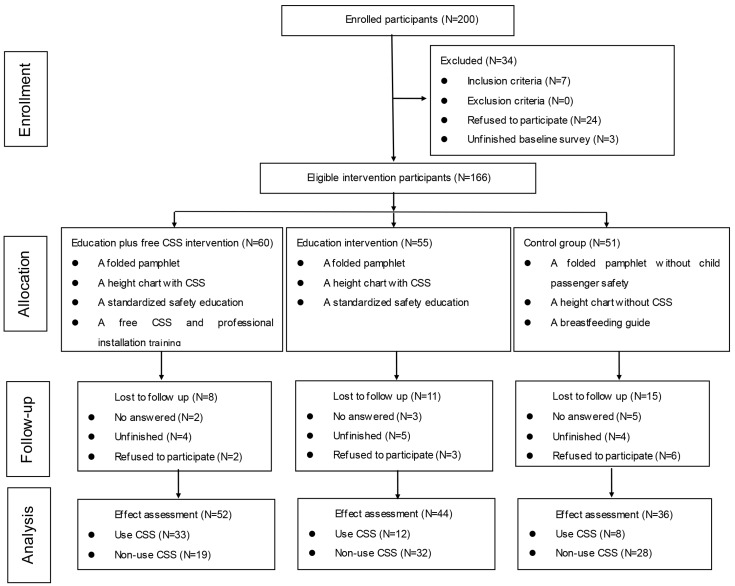
Study procedures.

Two hundred parents of newborns in the two hospitals during the research period were introduced to the research and invited to participate in the study. Of these, 166 parents agreed and were enrolled in the study, while 76 were excluded (24 refused to participate in the study, 43 were not eligible, and nine had incomplete baseline surveys) (see Figure 1). Of 166 enrolled parents, 60 were in the education plus the free CSS intervention group, 55 were in the education intervention group and 51 were in the control group. Three months after the interventions, 52 parents who remained in the education plus the free CSS intervention group, 44 in the education intervention group and 36 in the control group completed the follow-up surveys. Of 34 parents who were lost to follow-up, 10 were unable to contact, 11 refused to participate and 13 had incomplete telephone follow-up (see Figure 1).

No significant differences were observed in demographic characteristics among the three groups (see Table 1). The average age of parents in the education plus the free child safety seat (CSS) intervention group, education intervention group and control group were 28.13 ± 3.766, 28.34 ± 4.856 and 28.17 ± 3.365 years old, respectively, with no significant difference (F = 0.315, *p* = 0.731). More than half of the participating parents were female and received university or higher education. About 53.85% of participating parents self-reported that their household income was more than 4,000 Yuan per month. Over one-third (35.95%) of fathers of newborns were businessmen, and 38.43% of mothers were housewives.

### 3.2. Changes in the Use of CSS after the Intervention

When comparing the changes in CSS use among the three groups after the intervention, the 3 × 2 table chi-square test was conducted by SPSS 19.0 software (SPSS 19.0, SPSS Inc., Chicago, IL, USA), and the results showed statistically significant differences in CSS utilization among three groups (χ^2^ = 19.6109, *p* = 0.000) (see Table 2). Further comparisons were made to determine which two groups were different, using adjusted for the Alpha value (according to the calibration standard formula ***α****'* = ***α***/2(**κ** − 1), where ***α*** was 0.05; and **κ** was the number of sample size, ***α'*** = 0.05/2 (3 − 1) = 0.0125. There was a significant difference in CSS use when comparing education plus free CSS intervention group with education only intervention group (χ^2^ = 12.534, *p* = 0.000) or the control group (χ^2^ = 14.539, *p* = 0.000). However, differences between the education only intervention group and the control group were not significant (χ^2^ = 0.269, *p* = 0.796). The results suggest that the education intervention alone did not have significant effect in improving the use of CSS. However, when the education intervention was combined with a free CSS and professional installation training, it had a significant effect on increased CSS use.

### 3.3. Changes in Child Passenger Safety Knowledge and Risky Driving Behaviors after Intervention

A general safety knowledge score was constructed to test the differences among the three groups after the intervention. There were seven knowledge items. Each correct item was given a score of one, with the highest total score possible being seven for everyone. The average score of the education plus free CSS intervention group, the education only group and the control group were 45.714 ± 9.178, 32.857 ± 10.574, 24.000 ± 8.756, respectively, with statistically significant differences (F = 9.180, *p* = 0.002). Afterwards, for the Alpha value, the results showed that there was a significant increase in parental knowledge on child passenger safety between the education plus free CSS intervention group and the control group after the intervention (*p* = 0.001, 95% CI: 8.2643–35.1643). No differences existed between any two groups (*p* > 0.005).

Significantly increased knowledge after the intervention was observed in the education plus free CSS intervention group, regarding the protective effect of seat belts (χ^2^ = 13.1680, *p* = 0.0003) and airbags (χ^2^ = 51.0545, *p* = 0.0000), danger of holding baby passengers on an adult’s lap (χ^2^ = 65.8471, *p* = 0.0000) and necessity of CSS legislation (χ^2^ = 10.0838, *p* = 0.0015) (see Table 3). The proportion of drivers wearing a safety belt increased from 90% to 100% (χ^2^ = 5.2525, *p* = 0.0219) and answering a phone without a device decreased from 29% to 4% (χ^2^ = 11.8837, *p* = 0.0006). In the education only group, knowledge about the protective role of the airbag (χ^2^ = 5.8667, *p* = 0.0154), and CSS (χ^2^ = 5.4363, *p* = 0.0197) increased significantly (see Table 3). The proportion of drivers wearing seat belts increased from 66% to 86% (χ^2^ = 5.0661, *p* = 0.0244). In the control group, there were no significant changes except the statistically significant increase in knowledge about CSS (χ^2^ = 4.4308, *p* = 0.0353) (see Table 3). In addition, 64 out of 132 (48.5%) participants identified danger of holding a baby passenger on the adult lap before the intervention. After the intervention, despite the fact that knowledge of child passenger safety had improved, 15.9% (21/132) of control group parents still believed it was safe to hold a baby on the lap, with most of them (95.2%) being CSS non-users.

### 3.4. Effects of the Intervention on Child Passenger Safety Knowledge, Risky Driving Behaviors, and CSS Use

The results from logistic regression showed that education plus free CSS group had a significant increase in child passenger safety knowledge on airbags {odds ratio (OR) = 8.4640, 95% confidence interval (CI): 1.5410–46.4730}, feeding baby in a driving car (OR = 0.0300, 95% CI: 0.0020–0.3680) and CSS laws (OR = 0.0190, 95% CI: 0.0030–0.1110) compared to the control group. The education only group had statistically higher knowledge about CSS laws (OR = 4.1570, 95% CI: 0.9250–18.6800) as compared to the control group (see Table 4). Except for using a mobile phone while driving, no statistically significant differences were observed on risky driving behaviors among the three groups.

## 4. Discussion

This study evaluated the effect of education plus free CSS intervention strategies in improving child passenger safety knowledge and use of child safety seats among newborn parents in maternity departments. The results showed that the education plus free CSS intervention was effective in improving child passenger safety knowledge and use of CSS measured three months after the intervention. Specifically, in the education plus free CSS intervention group, the knowledge of safety belts, airbags, holding a baby in the vehicle and CSS legislation increased statistically after the intervention; drivers wearing safety belts increased from 90% to 100%, while those answering a phone without a device decreased from 29% to 4%. At the three-month follow-up, the proportion of CSSs used by participants in the education plus free CSS intervention group was significantly higher than use in the education only group and control group. These findings have demonstrated that an education about child passenger safety coupled with a free CSS and professional installation training was effective in improving child passenger safety knowledge and use of CSS.

While the education only group did not show a significant increase in CSS use, parents in the education only group had significantly increased knowledge on airbags and CSS after the intervention; the driver’s safety belt use increased from 66% to 86%. However, when compared with participants in the education plus free CSS intervention group, the effect in the education only group was less. These findings suggested that education without offering free CSS might be insufficient in improving CSS use.

Our results also indicated a lack of knowledge of parents of newborns in China about child passenger safety and CSS use for infants. Before the intervention, 48 parents of newborns, or 36.4% of the participants, thought that the seat belt could effectively protect child passengers. Of 132 participants, 100 parents of newborns, or 75.8% of the participants, reported the airbag could effectively keep a child passenger safe. Nearly half (48.5%) of participants thought that it was safe to hold a baby passenger on one’s lap. After the intervention, even though the knowledge of child occupant safety had improved, nearly 16% of parents still believed in the safety of holding a baby on one’s lap. Most of them were CSS non-users; only one participant used CSS because of long trips in which he/she was too tired to hold the baby all of the time. Over 30% of the parents said it was not necessary to enact a law for CSS. In China, the usage of CSS in metropolis was about 5%, while in a small city, such as Shantou where this study was conducted, use is less than 1%. Most child passengers are seated on the lap of an adult without any safety restraint system in the car, especially for newborn babies. Although our results suggest that education only was not as effective as education plus CSS to increase CSS use, education was found to have a significant role in improving parents’ child passenger safety knowledge, a predisposing factor for behavior change. Our results suggest that comprehensive education programs need to be developed to widely disseminate child passenger safety messages before a national law on child passenger safety is enacted.

Child passenger safety laws have been well implemented and enforced in the United States and other European countries for many years [7,10,22,23,24]. In the US, CSS laws have been enacted for more than two decades, and the usage of CSS was 98% among infants and 89% among children age 1 to 3 in 2006 [25]. However, in China, only two existing intervention studies have been published to date on knowledge and behaviors related to use of CSS [7,21]. Our previous observational study showed extremely low usage of CSS, with only 22 children wearing CSS among 3333 children passengers [2]. Our results from in-depth interviews with the parents of a child showed that a lack of awareness of CSS, poor perception, and no current mandatory law for CSS were the leading reasons for low use of CSS [26]. In 2014, the first local legislation on child passenger safety was implemented in Shanghai, which required that children younger than four years old wear a CSS [27]. Research in the United States has demonstrated the effectiveness of laws in increasing CSS use and protecting child passengers from injuries [9,28]. However, in Japan, a study that evaluated child passenger injuries before and after the CSS law did not show significant reduction in child passenger injuries because of inappropriate use [9]. Therefore, when evaluating the benefit of CSS legislation, it is crucial to quantify both CSS use and injury outcomes [29].

## 5. Limitations

This study has three limitations. First, there was a selection bias since the study used convenience sampling to recruit participants. The sample population also merely represented the parents of newborns who owned a car, which could not be generalized to all parents of newborns. To obtain a better representation of the participants, a larger scale intervention study among parents of kindergarten and primary school students should be conducted in a future study. Second, the long-term effect of the intervention cannot be measured. The effects were measured three months after the intervention, which might be too early to evaluate the true effect of the intervention. Babies traditionally stay home for the first three months. Because of this, even though some parents of newborns had a CSS, they may not have had a chance to use it. It would be ideal to have longer follow-up time. Finally, a total of 166 parents of newborns completed the intervention, but only 132 parents completed the three-month follow-up, with the rate of loss to follow-up being 20% (34/166). The reasons for loss to follow-up included no answer, unfinished questionnaire and refusal to participate. The attrition rate may lead to distortion of the study results. Without the data of participants who did not complete the three-month follow-up, the effect of intervention measures might be underestimated. On the other hand, in the control group, follow-up loss was greater than in both intervention groups, which may result in relative lower usage of CSS in the control group when comparing the three groups, which would exaggerate the effect of intervention measures in intervention groups. Additional methods to reduce loss to follow up should be included in future studies.

## 6. Conclusions

This study evaluated the effect of education plus free CSS, and education only intervention strategies on improving the newborn parent’s child passenger safety knowledge and use of CSS. An intervention combining child passenger safety education and free CSS and professional installation training was effective in improving the knowledge and behaviors of traffic safety among parents of newborns. This study’s findings call for more efforts including legislation to increase the use of CSS in order to protect children from traffic-related injuries and deaths.

## Figures and Tables

**Table 1:** Demographic characteristics of surveyed newborn parents among three groups.

Items	Education Plus Free CSS		Education Only		Control		χ^2^	*p-*Value
	N	R (%)	N	R (%)	N	R (%)		
Gender							2.8596	0.2394
Male	24	46.15	13	29.55	13	36.11		
Female	28	53.84	31	70.45	23	63.89		
Place of residence							2.5385	0.281
Shantou	34	65.38	33	75	21	58.33		
Other	18	34.62	11	25	15	41.67		
Newborn father’s degree of education							2.8357	0.5857 *
Primary school or below	1	1.92	0	0	1	2.78		
High school	21	40.38	24	54.55	17	47.22		
University or above	30	57.69	20	45.45	18	50		
Newborn mother’s degree of education							1.2889	0.8633 *
Primary school or below	2	3.85	2	4.55	1	2.78		
High school	22	42.31	23	52.27	18	50		
University or above	28	53.84	19	43.18	17	47.22		
Household per capita income							0.9955	0.6079
<¥4,000	24	46.15	24	54.55	16	44.44		
≥¥4,000	28	53.85	20	45.45	20	55.56		

* Fisher’s exact probability method.

**Table 2:** The usage of child safety seat (CSS) in three groups among parents of newborns.

Groups	The Usage of CSS		Total	χ^2^	*p-*Value
	Use n (%)	Non-Use n (%)			
Education plus free CSS	33 (62.2)	19 (24.0)	52	19.6109	0.0001
Education only	12 (22.6)	32 (40.5)	44		
Control	8 (15.1)	28 (35.4)	36		
Total	53	79	132		

**Table 3:** The pre- and post-intervention comparative results on child passenger safety knowledge among three groups.

Knowledge Items	Education Plus Free CSS		*p*-Value	Education Only		*p*-Value	Control		*p*-Value
	Pre	Post		Pre	Post		Pre	Post	
The car belt cannot effectively protect child passengers’ safety.	36	50	0.0003	28	36	0.0555	20	29	0.0229
The airbag cannot effectively protect child passengers’ safety.	12	48	0.0000	11	22	0.0154	9	15	0.1336
Holding baby passengers on the lap is unsafe.	9	50	0.0000	35	37	0.5804	24	24	1.0000 *
Feeding baby passengers in a driving car is unsafe.	47	50	0.2404	38	41	0.2912 *	32	35	0.1643 *
It is necessary for children under 4 years old to use CSS.	43	49	0.0655	33	41	0.0197	22	30	0.0353
The best suitable position for installing CSS is in the middle of the back.	8	25	0.0003	11	14	0.4782	7	10	0.4051
It is necessary enact a law to require use of CSS.	35	48	0.0015	32	39	0.1128	22	25	0.4577

* Fisher’s exact probability method.

**Table 4:** The results of binomial logistic regression on knowledge and behaviors ^a^.

Items	Groups ^b^	OR	95% CI		χ^2^	*p*-Value
The seat belt cannot effectively protect child passengers’ safety.	EP	1.9890	0.1720	23.0560	0.6678	0.4138
	EO	0.5650	0.1170	2.7200	1.1494	0.2837
The airbag cannot effectively protect child passengers’ safety.	EP	8.4640	1.5410	46.4730	5.6655	0.0173
	EO	1.2190	0.3740	3.9740	1.7254	0.1890
Holding baby passengers on the lap is unsafe.	EP	4.7400	0.2850	78.9660	0.2164	0.6418
	EO	6.3930	1.1970	34.1550	1.4014	0.2365
Feeding baby passengers in a driving car is unsafe.	EP	0.0300	0.0020	0.3680	6.8609	0.0088
	EO	0.2020	0.0250	1.6190	0.0368	0.8478
It is necessary for children under 4 years old to use CSS.	EP	5.6910	0.3520	91.9870	0.9943	0.3187
	EO	1.9200	0.2460	15.0080	0.0357	0.8501
The best suitable position for installing CSS is the middle of the back.	EP	1.9290	0.4710	7.9000	0.7345	0.3914
	EO	1.1830	0.3750	3.7320	0.0814	0.7754
It is necessary to enact a law to require use of CSS.	EP	0.0190	0.0030	0.1110	29.7910	<0.0001
	EO	4.1570	0.9250	18.6800	20.4490	<0.0001

^a^ Consider the control group as reference group; ^b^ EP, Education Plus Free CSS; EO, Education Only.
